# Modification by Influenza on Health Effects of Air Pollution in Hong Kong

**DOI:** 10.1289/ehp.11605

**Published:** 2008-10-03

**Authors:** Chit Ming Wong, Lin Yang, Thuan Quoc Thach, Patsy Yuen Kwan Chau, King Pan Chan, G. Neil Thomas, Tai Hing Lam, Tze Wai Wong, Anthony J. Hedley, J.S. Malik Peiris

**Affiliations:** 1 Department of Community Medicine and School of Public Health, University of Hong Kong, Hong Kong Special Administration Region, People’s Republic of China; 2 Department of Family and Community Medicine, Chinese University of Hong Kong, Hong Kong Special Administration Region, People’s Republic of China; 3 Department of Microbiology, University of Hong Kong, Hong Kong Special Administration Region, People’s Republic of China

**Keywords:** air pollution, cardiorespiratory disease, effect modification, hospitalization, influenza, mortality

## Abstract

**Background:**

Both influenza viruses and air pollutants have been well documented as major hazards to human health, but few epidemiologic studies have assessed effect modification of influenza on health effects of ambient air pollutants.

**Objectives:**

We aimed to assess modifying effects of influenza on health effects of ambient air pollutants.

**Methods:**

We applied Poisson regression to daily numbers of hospitalizations and mortality to develop core models after adjustment for potential time-varying confounding variables. We assessed modification of influenza by adding variables for concentrations of single ambient air pollutants and proportions of influenza-positive specimens (influenza intensity) and their cross-product terms.

**Results:**

We found significant effect modification of influenza (*p* < 0.05) for effects of ozone. When influenza intensity is assumed to increase from 0% to 10%, the excess risks per 10-μg/m^3^ increase in concentration of O_3_ increased 0.24% and 0.40% for hospitalization of respiratory disease in the all-ages group and ≥ 65 year age group, respectively; 0.46% for hospitalization of acute respiratory disease in the all-ages group; and 0.40% for hospitalization of chronic obstructive pulmonary disease in the ≥ 65 group. The estimated increases in the excess risks for mortality of respiratory disease and chronic obstructive pulmonary disease in the all-ages group were 0.59% and 1.05%, respectively. We found no significant modification of influenza on effects of other pollutants in most disease outcomes under study.

**Conclusions:**

Influenza activity could be an effect modifier for the health effects of air pollutants particularly for O_3_ and should be considered in the studies for short-term effects of air pollutants on health.

The deleterious effects of air pollution on human health have been well studied. Numerous studies have demonstrated that air pollution is associated with respiratory and cardiovascular diseases (CVDs) (Health Effects Institute 2004; Samet et al. 2000; Spix et al. 1998). Similarly, influenza also contributes a heavy burden of morbidity and mortality from respiratory diseases (RDs) and CVDs (Chow et al. 2006; Thompson et al. 2003, 2004; Wong et al. 2004, 2006). The effects of air pollutants and influenza viruses on disease pathogenesis could potentially interact synergistically because both of them affect the respiratory system, from the nasal cavity and nasopharynx to the main airways and alveoli, and also through systemic effects on the cardiovascular system. The transmission of influenza viruses is believed to rely on short-distance dispersion of droplets. However, there is speculation that ambient air pollutants, especially particulate matter ≤ 10 μm in aerodynamic diameter (PM_10_), may facilitate the spread of influenza viruses by providing condensation nuclei for the virus droplets. This is believed to be critical to long-range transmission of influenza viruses (Hammond et al. 1989).

Laboratory studies have provided some evidence to support interaction between influenza and air pollution on their adverse effects on human health. As early as the 1970s, a series of experiments in mice infected by influenza viruses showed an increased incidence of pneumonia after exposure to sulfur dioxide (Fairchild et al. 1972). A more recent study showed that exposure to diesel exhaust, an important source of PM_10_, could generate oxidative stress in human nasal and bronchial epithelial cells and could also enhance the attachment of influenza virus to these cells (Jaspers et al. 2005). However, the relevance of these experimental studies is uncertain in that the concentrations of pollutants in animal and human laboratory studies were 10–1,000 times higher than the average ambient levels observed in even relatively highly polluted locations such as Hong Kong. Population-based studies are needed to assess the effects of human exposure in the community.

Despite numerous attempts to study the biologic mechanisms behind the interaction between air pollutants and influenza viruses, to date only the confounding effect of influenza has been considered in the short-term effects of air pollution on human health in several multicity projects (Katsouyanni et al. 2001; Touloumi et al. 2005). In our study we evaluated such an interaction from an epidemiologic perspective. This study is part of the Public Health and Air Pollution in Asia (PAPA) project (Health Effects Institute 2006), which involves four Asian cities and aims to provide insight on the short-term effects of air pollution on mortality and hospitalization. In particular, we examined four major ambient air pollutants in Hong Kong: SO_2_, nitrogen dioxide, ozone, and PM_10_. Specifically, we assessed effect modification of influenza on air-pollution–associated hospitalization for RD and its subcategories acute respiratory disease (ARD) and chronic obstructive pulmonary disease (COPD) and on CVD, as well as on air-pollution–associated mortality of RD, COPD, and CVD.

## Materials and Methods

### Sources of data

We retrieved daily counts of hospitalization from the 14 general hospitals of the Hospital Authority of Hong Kong for the period 1996–2002. We based these counts on discharge diagnoses of admission records coded according to the *International Classification of Diseases, 9th Revision* (ICD-9) (World Health Organization 1977) and *10th Revision* (ICD-10) (World Health Organization 1994). The selected disease categories included CVD (ICD-9 codes 390–459; ICD-10 codes I00–I99) and RD (ICD-9 codes 460–519; ICD-10 codes J00–J98), and subcategories of COPD (ICD-9 codes 490–496; ICD-10 codes J40–J47) and ARD (ICD-9 codes 460–466, 480–487; ICD-10 codes J00-J05). We divided these data into three age groups [all ages combined, ≥ 65 years of age, and 0–14 years of age (for ARD)] and three sex groups (males and females and combined). We obtained the daily numbers of deaths for the above disease categories over the study period from the Census and Statistics Department of Hong Kong. Because of relatively small numbers of daily mortality, we did not perform the age- and sex-stratified analyses when assessing the effect modification of influenza on the effects of air pollution on mortality of RD, COPD, and CVD.

Daily mean temperature and relative humidity were provided by the Hong Kong Observatory (Hong Kong Observatory 2005). We obtained air pollutant concentrations from eight monitoring stations from the Hong Kong Environmental Protection Department (HKEPD). We derived daily 24-hr mean concentrations of NO_2_, SO_2_, and PM_10_ and 8-hr (1000–1800 hours) mean concentration of O_3_ from the HKEPD database. We defined daily concentrations as nonmissing if at least 18 of 24 hourly concentrations of NO_2_, SO_2_, and PM_10_, and six of eight hourly concentrations of O_3_ were available. We first centered non-missing daily means for each station *i* [i.e., we subtracted individual daily concentrations (*X_ij_*) by an annual station mean (*X_i_*) for each day *j*]. We then combined the centered data from all stations and added them into the annual mean of all stations (*X*) to form *X*′*_ij_* = (*X_ij_* − *X_i_* + *X*). We computed the daily (mean) concentrations of individual pollutants for analysis by taking the mean of centered *X*′*_ij_* over all stations (Wong et al. 2001).

We obtained weekly numbers of specimens positive for influenza viruses A and B (Flu A+B) or respiratory syncytial virus (RSV) and total numbers of specimens tested from the Microbiology Laboratory of Queen Mary Hospital (Hong Kong). This laboratory routinely collects the respiratory specimens from the patients with influenza-like symptoms (fever > 38°C, with cough and/or sore throat) over the Hong Kong Island and conducted the immunofluorescent antigen test for Flu A+B. We derived the proportions of positive isolates of Flu A+B and RSV from the total specimens tested, which we then used to assess the effects of influenza and to adjust for the potential confounding effect of RSV in the model. The proportion of positive isolates of Flu A+B, which is defined as influenza intensity in this study, has been used in assessment of influenza effects on hospitalization and mortality (Wong et al. 2004, 2006). We used the proportions of specimens positive for influenza as a continuous measure for influenza activity instead of defining influenza epidemics. This allowed us to avoid the bias potentially posed by unpredictable seasonality of influenza in Hong Kong (Yang et al. 2008). We assumed constant activity of influenza virus and RSV within a week and interpolated the weekly proportions to the daily data, which we set the same from Sunday to Saturday.

### Data analysis

We used generalized additive modeling in this study (Hastie and Tibshirani 1990). To control for potential confounding factors due to time-varying covariates, we first built the core model on the daily counts of hospitalization or mortality for each disease category, with dummy variables for the day of the week and public holidays to control for the variation associated with these factors. We controlled the weather conditions by adding into the core model natural spline smoothing functions of daily mean temperature and relative humidity, each with three degrees of freedom. Additional adjustments in the core model included natural spline smoothing for time trend and seasonality. We chose the degrees of freedom for each smoothing function according to the observed autocorrelations of the residuals using partial autocorrelation function (PACF) plots. To reduce the autocorrelations that could not be minimized through smoothing, we also added autoregressive terms into the core model. For the core models fitted to the mortality data, we considered the time-varying confounding factors as adequately controlled if the absolute values of PACF coefficients were < 0.1 for the first 2 lag days and there were no systematic patterns in the PACF plots. Because of the large autocorrelations within the hospitalization data, we added autoregressive terms until all the PACF coefficients at the first 30 lag days were < 0.1.

After the core model for each health outcome had been established, we entered single air pollutant concentrations, influenza intensity, and the interaction term defined by the cross-product between these two variables into the core model to obtain the inter action model. To adjust for lag effects, we used the concentrations of air pollutants averaged over the current day and the previous day (lag 0–1). We added RSV proportions in order to control for the potential confounding of RSV with influenza. By assuming that the influenza intensity of the same day was equal to zero, we calculated from the interaction model the excess risk (ER) of hospitalization or mortality per 10 μg/m^3^ increase of each air pollutant as the baseline effect of air pollution. We also calculated the ER per 10-μg/m^3^ increase of each air pollutant when we assumed the influenza intensity to be its mean level of 10%, and the change in ER for each air pollutant associated hospitalization or mortality (a measure for the modifying effect of influenza) from the interaction model, under the assumption that the influenza intensity increased from 0% to 10%. We completed all the statistical analyses using the statistical software package R version 2.5.1 with mgcv package version 1.3–25 (R Foundation for Statistical Computing, Vienna, Austria).

## Results

During the study period of 1996–2002, influenza intensity had an average of 10.1% and ranged from 0% to 54.6%. The average concentrations of air pollutants were 58.7, 17.8, 51.6, and 36.9 μg/m^3^ for NO_2_, SO_2_, PM_10_, and O_3_, respectively (Table 1). Table 1 also shows the summary statistics for meteorologic data as well as daily mortality and hospitalization outcomes. On average, mortality counts of CVD were higher than those of RD, but for hospitalization it was the opposite (Table 1).

The baseline effects of the air pollutants on mortality, which we calculated under the assumption of zero influenza intensity, were statistically significant (*p* < 0.05) for CVD associated with NO_2_ and SO_2_, and for RD associated with NO_2_ (ER, 1.23%, 1.64%, and 1.24%, respectively) but were not significant (*p* > 0.05) for all mortality outcomes associated with O_3_ (Table 2). We found significant modifying effects of influenza for the O_3_ effects on mortality for RD and COPD (ER increased 0.59% and 1.05%) but not for the other pollutants.

The effects of NO_2_ on hospitalization at the baseline level of influenza were statistically significant (*p* ≤ 0.05) for all the health outcomes under study (except for ARD), with ER ranging from 0.85% to 1.84%. We found significant (*p* ≤ 0.05) modifying effects of influenza for the NO_2_ effects on hospitalization of COPD in the groups ≥ 65 years of age (ER increased 0.43%; Table 3).

We found significant (*p* ≤ 0.001) effects of SO_2_ on hospitalization of CVD at the baseline level of influenza (ER, 1.1% and 1.5% for all-ages and ≥ 65 age groups). We found modifying effects of influenza for the effect of SO_2_ on hospitalization to be significant (*p* ≤ 0.05) only for ARD in all-ages group with ER increased 0.86% (Table 3).

For PM_10_, we found significant effects (*p* ≤ 0.05) at the baseline level of influenza for all the health outcomes under study (ER ranging from 0.55% to 1.49%). We found no significant modifying effects of influenza for PM_10_ on hospitalization in all the disease categories under study (Table 4).

We found significant effects of O_3_ at the baseline level of influenza (*p* ≤ 0.01) for all RD groups under study (ER ranged from 0.53% to 1.39%), but not for CVD disease. Changes in ER associated with O_3_ for hospitalization of RD were significant (*p* ≤ 0.05). ER increased 0.24% and 0.40% for RD in all-ages and ≥ 65 age groups, respectively, and increased 0.46% for ARD in all-ages and 0.40% for COPD in ≥ 65 age group when influenza intensity increased from 0 to 10% (Table 4).

In analyses stratified by males and females, we found significant effect modification of influenza (*p* ≤ 0.05) for the effects of O_3_ on hospitalization, with increases of 0.38% and 0.48% in ER for RD in both males and females in the ≥ 65 age groups, respectively; and with increase of 0.63% and 0.88% in ER for COPD in the females of all-ages and ≥ 65 groups, respectively. The effect modifications of influenza on hospitalization associated with O_3_ tend to be higher and more significant for females than for males (Table 5). We found no significant effect modification of influenza for effects of the other pollutants on hospitalization in most health outcomes under study, except for NO_2_ with an increased ER on COPD in the males in the ≥ 65 group, and for PM_10_ with decreased ER on RD in the males in the all-ages group [data not shown; see Supplemental Material, Tables 1–3 (http://www.ehponline.org/members/2008/11605/suppl.pdf)].

## Discussion

Previous studies have identified modifying effects of climates for the short-term effects of air pollutants on mortality; for example, the effects of air pollutants were higher in the warm season (Katsouyanni et al. 1997), but other reports found no noticeable changes between seasons (Schwartz 2000) or even found larger effects in the cool season (Wong et al. 2002). Other effect modifiers have been identified as well, such as NO_2_ concentration, which markedly modified the effects of PM_10_ on mortality (Katsouyanni et al. 2001). Our study demonstrated effect modification by influenza on the effects of single air pollutants, particularly O_3_, both on mortality and on hospitalization. The difficulty in assessing the interaction between influenza and air pollutants at the population level may occur partly because their seasonal peaks do not overlap. For example, in Hong Kong NO_2_ and PM_10_ are highest in December and January, SO_2_ is highest around August, and O_3_ peaks from September through December (data not shown), and influenza tends to reach peaks in January–February and May–July (Wong et al. 2004). Taking advantage of long-standing surveillance for air pollutants and influenza virus activity, we estimated the extent to which influenza modifies the effects of air pollutants in this study.

Interestingly, our results suggest that positive effect modification of influenza on the effects of O_3_ might be more pronounced compared with its modification of the impact of the other three pollutants. O_3_ is a strong oxidative agent and also a potent pulmonary irritant. For effects of air pollution on mortality, we found significant increases in influenza intensity only in the effect of O_3_ on RD and COPD; this result did not change the effects of the other air pollutants. For effects of air pollution on hospitalization, effect modification of influenza on the effects of O_3_ could be detected for ARD, RD, and COPD. However, for the other three pollutants, we detected such modification only for one of the three RD categories (i.e., NO_2_ for COPD, SO_2_ for ARD, and PM_10_ for RD). Several laboratory-based studies have demonstrated the positive interaction between effects of O_3_ and influenza. In a study using mice, continuous exposure of 0.5 ppm (1.0 mg/m^3^) O_3_ attenuated acute lung damage during early influenza infection but, conversely, exacerbated long-term lung injury (Jakab and Bassett 1990). Studies on respiratory viruses other than influenza also suggested a possible synergistic effect of O_3_ on respiratory infection. For example, exposure to 0.2 ppm (0.4 mg/m^3^) O_3_ has been reported to increase the efficiency of rhinovirus infection of respiratory epithelial cells from 41% to 67%, and this effect could be attenuated by the use of anti-oxidants (Spannhake et al. 2002). Exposure to 0.2 ppm O_3_ markedly increased adhesion of polymorphonuclear leukocytes (PMNs) to human tracheal epithelial cells (Tosi et al. 1994). However, another study in young male volunteers showed that intermittent exposure to 0.3 ppm O_3_ after infection by rhinovirus did not change adhesion of PMNs to the nasal epithelium, or levels of interferon (Henderson et al. 1988). Interaction between influenza and O_3_ needs more support from laboratory studies.

Although PM_10_ is associated with significant risk of hospitalization for almost all the health outcomes under study at both the baseline and mean level of influenza intensity, we identified significant negative interaction only between PM_10_ and influenza in their effects on hospitalization for RD in males in the all-ages group. Influenza intensity was associated with reduction of ER of PM_10_. This negative interaction is in contrast to many previous studies, which have demonstrated that exposure to PM_10_ increased the host susceptibility to influenza infection by up-regulating immuno-regulatory cytokines (Diaz-Sanchez 1997), by depressing pulmonary macrophage function (Yin et al. 2002) and also by increasing virus attachment to respiratory epithelial cells (Jaspers et al. 2005). We postulate that the seemingly negative interaction between effects of PM_10_ and influenza on hospitalization observed in this study may lie in the linkage of ultraviolet radiation (UVR) to PM_10_. In Hong Kong, concentrations of PM_10_ are negatively correlated with intensity of UVR in the ambient air (*r* = −0.17, *p* < 0.001). When the level of PM_10_ concentration is high, exposure to UVR is low. Exposure to erythemal doses of UVR before influenza infection has been shown to increase the influenza-associated mortality in mice in a dose-dependent manner, without remarkably changing immunity response against influenza (Ryan et al. 2000). The adverse health effects of PM_10_ may be partially attenuated by the reduced effects due to the associated lowered levels of UVR. This attenuation may contribute to the observed significant negative interaction between PM_10_ and influenza, which we found for respiratory hospitalization for males [see Supplemental Material, Table 3 (http://www.ehponline.org/members/2008/11605/suppl.pdf)], although both PM_10_ and influenza infection could independently increase the risk of hospitalization. However, the results of the laboratory studies describing the effects of UVR on immune function remain controversial and uncertain, and it was suggested that severity of respiratory viral infections could be related to radiation doses from UVR [reviewed by Cannell et al. (2006)]. Future studies may provide more insight into the role of UVR in interaction between influenza and PM_10_.

In this study, we found significant positive interaction between the effects of influenza and NO_2_ on hospitalization for COPD in the males in the ≥ 65 age group. Some laboratory studies described a positive interaction between influenza and NO_2_ (Chauhan et al. 2003; Frampton et al. 1989; Spannhake et al. 2002), whereas others did not detect such an interaction (Devlin et al. 1999; Frampton et al. 2002; Goings et al. 1989). We also found that the increase in influenza intensity would significantly raise the effects of SO_2_ on hospitalization for ARD. The damage caused by SO_2_ to the human pulmonary defense system mainly involves nonspecific airway reactivity, such as lower mucociliary transportation rate, decreased alveolar clearance of deposited particles, and pulmonary macrophage function (Schlesinger 1999). People with hypersensitive airways, including COPD patients (Cazzola et al. 1991), have been shown to be more sensitive to SO_2_ than are healthy individuals (Schlesinger 1999), but we observed the marked positive interaction between SO_2_ and influenza only in ARD, and not in COPD or CVD.

Interaction between air pollutants and influenza could have a competitive or a saturation effect, in that both need to attach to respiratory epithelial cells to cause harm. Therefore, if one factor saturates the sites on a particular pathway, there may be little room for the other to exert any additive effect. Early studies in mice showed that exposure to O_3_ (0.9 ppm for 3 hr) and SO_2_ (6 ppm for 7 days) could almost completely or partially inhibit influenza virus growth in the nasal cavity (Andersen et al. 1977; Fairchild 1977). Similarly, a high dose of NO_2_ (1.5 ppm) could suppress the replication and release of RSV in human bronchial epithelial cells (Becker and Soukup 1999). A small reduction in the number of pneumonia cases was also observed after influenza-infected mice were exposed SO_2_ at a low concentration (of < 10 ppm) (Fairchild et al. 1972). Despite the increased awareness of the adverse impacts of both air pollution and influenza on human health, population-based studies on the potential synergistic effect or even competitive relationship between air pollutants and influenza viruses are lacking. Laboratory studies are insufficient to demonstrate a plausible mechanism for an interaction between influenza and air pollution on human health, and epidemiologic studies that can give support to formulation of plausible hypotheses at population level are needed.

The estimated effects of air pollutants at the mean level of influenza intensity are consistent with our previous studies for the short-term effects of air pollutants without adjustment for influenza intensity (Wong et al. 2001, 2002). In our study, the modifying effects estimated from the interaction models, in terms of changes in ER per 10-μg/m^3^ increase in concentration of an air pollutant when influenza intensity increased by 10%, say, for O_3_ ranged from 0.2% to 0.9%. These are substantial changes compared with the magnitude of air pollution effects of around 1% estimated in most studies (Wong et al. 2002, 2004). Our results show that the effects of air pollution (particularly O_3_) varied to some extent depending on the level of influenza virus in the circulation. We also did the analysis without adjustment for effect modification of influenza. Most estimates for the effects of air pollutants were significant, with levels lying between those in the absence of influenza activity and at the mean level of influenza intensity (data not shown). Environmental protection and other public health agencies should take influenza peak seasons into account when they issue air pollution and health warnings to the public.

We detected no significant modifying effects of influenza on the effects of air pollutants on mortality, except for O_3_ effects on mortality from RD and COPD. CVD was the only disease group of mortality and hospitalization in which we detected no significant interaction between influenza and any air pollutant, although influenza and air pollutants have been documented to be associated with CVD in several epidemiologic studies. Influenza is believed to accelerate the coagulation process by evoking proinflammatory response, stimulating prothrombotic cytokines, and increasing plasma viscosity in human (Madjid et al. 2004). The biologic impacts of ambient air pollutants on progression of CVDs have been frequently reported, especially for PM_10_ and O_3_. Similar to influenza, these air pollutants are believed to stimulate systematic inflammation and to increase oxidant stress, which may subsequently provoke cardiovascular events (Brook et al. 2004). However, interaction between influenza and ambient air pollutants for effects on cardiovascular mortality and hospitalization does not appear to be of sufficient strength to be detected in our model.

A limitation in the interpretation of these observations is that it is implausible to reliably separate the effects of air pollutants because they frequently react with each other; for example, NO_2_ can be photolyzed into nitric oxide and oxygen atoms, the latter of which combine with molecular oxygen to form O_3_ (Brook et al. 2004). Future studies for possible interaction between influenza and combined air pollution may provide more insight into the mechanisms by which RDs and CVDs associated with exposure to ambient air pollution are enhanced or weakened by influenza infection. A further limitation in interpretation of the results relates to multiple comparisons. With 24 health outcomes in hospitalization (including analyses by sexes) to assess for each pollutant, we expect to find about one statistically significant interaction result each at a 5% level. Thus, in the one significant effect modification we observed for SO_2_ or PM_10_, and the two significant effect modifications we observed for NO_2_ only with COPD (in males, and males and females combined in the ≥ 65 age group), we cannot exclude that the results may simply be attributable to chance for these two pollutants. For O_3_, we found eight statistically significant interactions, and two of them are significant at the 0.1% level. We believed that the results are unlikely to be attributable solely to chance at the 5% level even with Bonferroni adjustment.

In this study, we studied the impact of influenza on health effects of air pollution at lag 0–1 days. These lag days were recommended and chosen *a priori* for presentation of the main results in the PAPA studies. However, when considering the impact of influenza on health effects of air pollutant at longer lag days, we observed more statistical significant estimates for modifying effects of influenza on hospitalization outcomes (data not shown). We did not adjust for potential changes in mortality counts in year 2001 due to a switch of mortality codes from ICD-9 to ICD-10. Unfortunately, because the data were collected through a computerized system during the routine medical practice, we were unable to do the sensitivity analysis to assess the impact of switching codes on our results. However, because the changes in mortality counts were estimated to be small (Tsang and Cheung 2005), and because they affected only a short time period in the middle of the study period, we believe the impacts of inconsistent ICD codes on the results would be minimal.

## Conclusions

In this epidemiologic study of interactions between air pollution and influenza activity, we have shown that influenza modifies the effects of air pollution on mortality and hospitalization of RDs. These results support a link between air pollution mainly for O_3_ and influenza activity in their effects on human health, which have been demonstrated in animal studies. Further research is needed to clarify what conditions produce observable interactions between effects of air pollution and influenza and to provide the evidence for causality.

## Correction

In Table 1, the values for ARD were incorrect in the manuscript originally published online. They have been corrected here.

## Figures and Tables

**Table 1:** Summary statistics of influenza, air pollution, and meteorologic data.

			Percentile			
Variables	Mean ± SD	Minimum	25th	50th	75th	Maximum
Temperature (°C)	23.7 ± 4.7	8.9	19.6	24.7	27.8	31.7
Humidity (%)	78 ± 7.8	40.6	74.7	79.8	83.3	92
Influenza (%)	10.1 ± 10.1	0	2.2	7.2	14.5	54.6
Air pollutant (μg/m^3^)						
NO_2_	58.7 ± 20	10.1	45.1	56.3	69.6	168
SO_2_	17.8 ± 12.1	1.8	9.6	14.7	22.1	109.4
PM_10_	51.6 ± 25.3	13.5	31.8	45.5	66.7	188.5
O_3_	36.9 ± 23	−8.2^a^	19.2	31.7	50.8	196.6
Mortality, all ages (no.)						
RD	16.2 ± 5.2	3	12	16	19	34
COPD	5.9 ± 2.9	0	4	6	8	19
CVD	23.8 ± 6.5	6	19	23	28	54
Hospitalization, all ages (no.)						
RD	270.3 ± 56.3	143	230	266	303	586
ARD	104.9 ± 29.8	48	84	99	122	275
COPD	91.5 ± 20	41	77	89	104	176
CVD	203.5 ± 48.5	75	166	202	241	345
Hospitalization (no.)						
RD, ≥ 65 age group	138.5 ± 36.7	57	112	135	160	302
ARD, 0–14 age group	60.1 ± 19.5	22	46	56	72	149
COPD, ≥ 65 age group	59.6 ± 16.7	19	48	58	70	124
CVD, ≥ 65 age group	130.8 ± 30.1	47	108	128	152	222

a Value for O_3_ concentration is negative because air pollutant concentration data were standardized using the centering method.

**Table 2:** Modifying effects of influenza for the four pollutant effects on mortality.

		Modifying effect^a^			Baseline effect^b^			Effect at mean level of influenza intensity^c^		
Pollutant	Disease	ER	95% CI	*p*-Value	ER	95% CI	*p*-Value	ER	95% CI	*p*-Value
NO_2_	RD	0.18	−0.45 to 0.82	NS	1.24	0.27 to 2.22	*	1.42	0.45 to 2.41	**
	COPD	1.01	−0.03 to 2.05	NS	0.26	−1.34 to 1.87	NS	1.26	−0.33 to 2.89	NS
	CVD	0.15	−0.39 to 0.70	NS	1.23	0.41 to 2.06	**	1.39	0.56 to 2.22	#
SO_2_	RD	0.05	−1.12 to 1.23	NS	1.20	−0.38 to 2.81	NS	1.25	−0.35 to 2.88	NS
	COPD	0.32	−1.58 to 2.27	NS	−0.03	−2.67 to 2.69	NS	0.30	−2.35 to 3.01	NS
	CVD	−0.45	−1.45 to 0.55	NS	1.64	0.27 to 3.02	*	1.18	−0.20 to 2.57	NS
PM_10_	RD	0.08	−0.39 to 0.55	NS	0.69	−0.10 to 1.49	NS	0.77	−0.01 to 1.56	NS
	COPD	0.50	−0.26 to 1.27	NS	−0.05	−1.36 to 1.28	NS	0.45	−0.83 to 1.75	NS
	CVD	0.25	−0.15 to 0.65	NS	0.45	−0.23 to 1.13	NS	0.70	0.03 to 1.37	*
O_3_	RD	0.59	0.04 to 1.14	*	−0.16	−1.00 to 0.69	NS	0.42	−0.44 to 1.29	NS
	COPD	1.05	0.17 to 1.93	*	−0.11	−1.51 to 1.32	NS	0.94	−0.49 to 2.38	NS
	CVD	0.04	−0.42 to 0.51	NS	0.58	−0.15 to 1.31	NS	0.62	−0.12 to 1.36	NS

Abbreviations: CI, confidence interval; NS, not significant.

a Change in ER per 10-μg/m^3^ increase in concentration of air pollutant averaged over the lag 0 and lag 1 days when influenza intensity changed from the baseline (0%) to the mean level (10%).

b ER per 10-μg/m^3^ increase in concentration of air pollutant averaged over the lag 0 and lag 1 days when influenza intensity is assumed equal to 0.

c ER per 10-μg/m^3^ increase in concentration of air pollutant averaged over the lag 0 and lag 1 days when intensity is assumed equal to 10% (mean level of influenza intensity).

* 0.01 < *p* ≤ 0.05;

** 0.001 < *p* ≤ 0.01;

# *p* ≤ 0.001.

**Table 3:** Modifying effects of influenza for NO_2_ and SO_2_ effects on hospitalization.

			Modifying effect^a^			Baseline effect^b^			Effect at mean level of influenza intensity^c^		
Pollutant	Disease	Age group (years)	ER	95% CI	*p*-Value	ER	95% CI	*p*-Value	ER	95% CI	*p*-Value
NO_2_	RD	All	−0.09	−0.32 to 0.15	NS	0.85	0.51 to 1.18	#	0.76	0.51 to 1.01	#
		≥ 65	−0.07	−0.35 to 0.22	NS	1.06	0.64 to 1.48	#	0.99	0.68 to 1.30	#
	ARD	All	0.33	−0.03 to 0.70	NS	0.55	−0.02 to 1.12	NS	0.88	0.46 to 1.31	#
		0−14	−0.18	−0.57 to 0.22	NS	0.44	−0.16 to 1.04	NS	0.26	−0.19 to 0.71	NS
	COPD	All	0.09	−0.26 to 0.44	NS	1.84	1.32 to 2.35	#	1.93	1.54 to 2.32	#
		≥ 65	0.43	0.05 to 0.81	*	1.19	0.62 to 1.76	#	1.62	1.19 to 2.06	#
	CVD	All	0.04	−0.20 to 0.28	NS	0.98	0.63 to 1.33	#	1.02	0.76 to 1.29	#
		≥ 65	−0.03	−0.32 to 0.26	NS	1.30	0.89 to 1.70	#	1.27	0.96 to 1.57	#
SO_2_	RD	All	−0.24	−0.65 to 0.16	NS	0.31	−0.23 to 0.86	NS	0.07	−0.30 to 0.44	NS
		≥ 65	−0.33	−0.83 to 0.17	NS	0.37	−0.30 to 1.05	NS	0.04	−0.42 to 0.50	NS
	ARD	All	0.86	0.20 to 1.53	*	−0.77	−1.69 to 0.16	NS	0.09	−0.55 to 0.73	NS
		0–14	0.53	−0.16 to 1.22	NS	−0.60	−1.56 to 0.37	NS	−0.07	−0.74 to 0.60	NS
	COPD	All	0.35	−0.32 to 1.02	NS	0.29	−0.57 to 1.16	NS	0.64	0.04 to 1.25	*
		≥ 65	0.41	−0.29 to 1.13	NS	0.17	−0.77 to 1.12	NS	0.58	−0.07 to 1.24	NS
	CVD	All	−0.15	−0.60 to 0.30	NS	1.10	0.52 to 1.69	#	0.95	0.54 to 1.36	#
		≥ 65	−0.28	−0.80 to 0.25	NS	1.50	0.83 to 2.17	#	1.22	0.75 to 1.69	#

Abbreviations: CI, confidence interval; NS, not significant.

a Change in ER per 10-μg/m^3^ increase in concentration of air pollutant averaged over the lag 0 and lag 1 days when influenza intensity changed from the baseline (0%) to the mean level (10%).

b ER per 10-μg/m^3^ increase in concentration of air pollutant averaged over the lag 0 and lag 1 days when influenza intensity is assumed equal to 0.

c ER per 10-μg/m^3^ increase in concentration of air pollutant averaged over the lag 0 and lag 1 days when intensity is assumed equal to 10% (mean level of influenza intensity).

* 0.01 < *p* ≤ 0.05;

# *p* ≤ 0.001.

**Table 4:** Modifying effects of influenza for PM_10_ and O_3_ effects on hospitalization.

			Modifying effect^a^			Baseline effect^b^			Effect at mean level of influenza intensity^c^		
Pollutant	Disease	Age group (years)	ER	95% CI	*p*-Value	ER	95% CI	*p*-Value	ER	95% CI	*p*-Value
PM_10_	RD	All	−0.16	−0.34 to 0.01	NS	0.78	0.51 to 1.06	#	0.62	0.41 to 0.82	#
		≥ 65	−0.15	−0.36 to 0.06	NS	0.98	0.64 to 1.32	#	0.82	0.57 to 1.08	#
	ARD	All	0.13	−0.14 to 0.40	NS	0.70	0.24 to 1.16	**	0.83	0.48 to 1.18	#
		0–14	−0.11	−0.40 to 0.19	NS	0.55	0.06 to 1.03	*	0.44	0.08 to 0.81	*
	COPD	All	−0.14	−0.40 to 0.12	NS	1.49	1.06 to 1.92	#	1.34	1.02 to 1.67	#
		≥ 65	0.09	−0.19 to 0.37	NS	1.02	0.55 to 1.49	#	1.11	0.75 to 1.47	#
	CVD	All	−0.02	−0.20 to 0.16	NS	0.64	0.35 to 0.93	#	0.62	0.40 to 0.84	#
		≥ 65	−0.12	−0.33 to 0.09	NS	0.90	0.57 to 1.23	#	0.78	0.53 to 1.03	#
O_3_	RD	All	0.24	0.04 to 0.43	*	0.60	0.32 to 0.88	#	0.84	0.61 to 1.06	#
		≥ 65	0.40	0.16 to 0.64	#	0.53	0.17 to 0.89	**	0.93	0.64 to 1.22	#
	ARD	All	0.46	0.15 to 0.76	**	0.84	0.37 to 1.31	#	1.30	0.91 to 1.68	#
		0–14	−0.19	−0.51 to 0.13	NS	0.94	0.43 to 1.45	#	0.75	0.34 to 1.16	#
	COPD	All	0.17	−0.14 to 0.48	NS	1.39	0.93 to 1.85	#	1.56	1.18 to 1.93	#
		≥ 65	0.40	0.07 to 0.73	*	0.70	0.19 to 1.20	**	1.10	0.69 to 1.52	#
	CVD	All	0.20	−0.02 to 0.41	NS	0.00	−0.29 to 0.30	NS	0.20	−0.05 to 0.45	NS
		≥ 65	0.21	−0.03 to 0.46	NS	−0.03	−0.37 to 0.31	NS	0.18	−0.11 to 0.47	NS

Abbreviations: CI, confidence interval; NS, not significant.

a Change in ER per 10-μg/m^3^ increase in concentration of air pollutant averaged over the lag 0 and lag 1 days when influenza intensity changed from the baseline (0%) to the mean level (10%).

b ER per 10-μg/m^3^ increase in concentration of air pollutant averaged over the lag 0 and lag 1 days when influenza intensity is assumed equal to 0.

c ER per 10-μg/m^3^ increase in concentration of air pollutant averaged over the lag 0 and lag 1 days when intensity is assumed equal to 10% (mean level of influenza intensity).

* 0.01 < *p* ≤ 0.05;

** 0.001 < *p* ≤ 0.01;

# *p* ≤ 0.001.

**Table 5:** The baseline and modifying effects of influenza for effects of O_3_ on hospitalization by sex.

			Modifying effect^a^			Baseline effect^b^			Effect at mean level of influenza intensity^c^		
Disease	Sex	Age group (years)	ER	95% CI	*p*-Value	ER	95% CI	*p*-Value	ER	95% CI	*p*-Value
RD	Male	All	0.05	−0.18 to 0.27	NS	0.79	0.46 to 1.13	#	0.84	0.57 to 1.11	#
		≥ 65	0.38	0.08 to 0.69	*	0.66	0.20 to 1.11	**	1.04	0.68 to 1.41	#
	Female	All	0.22	−0.03 to 0.48	NS	0.65	0.26 to 1.04	**	0.87	0.56 to 1.19	#
		≥ 65	0.48	0.15 to 0.82	**	0.72	0.20 to 1.25	**	1.21	0.79 to 1.63	#
ARD	Male	All	0.01	−0.31 to 0.33	NS	1.03	0.52 to 1.54	#	1.03	0.63 to 1.44	#
		0–14	−0.27	−0.67 to 0.14	NS	0.95	0.31 to 1.59	**	0.68	0.17 to 1.19	**
	Female	All	0.05	−0.30 to 0.41	NS	0.58	0.01 to 1.15	*	0.63	0.18 to 1.09	**
		0–14	−0.19	−0.65 to 0.28	NS	1.23	0.49 to 1.98	**	1.04	0.45 to 1.64	#
COPD	Male	All	0.02	−0.32 to 0.36	NS	0.91	0.40 to 1.43	#	0.93	0.51 to 1.35	#
		≥ 65	0.25	−0.15 to 0.65	NS	0.71	0.10 to 1.32	*	0.96	0.47 to 1.46	#
	Female	All	0.63	0.19 to 1.07	**	1.55	0.87 to 2.23	#	2.19	1.63 to 2.75	#
		≥ 65	0.88	0.36 to 1.40	#	1.09	0.27 to 1.91	**	1.97	1.31 to 2.65	#
CVD	Male	All	0.21	−0.06 to 0.49	NS	−0.10	−0.49 to 0.30	NS	0.11	−0.21 to 0.44	NS
		≥ 65	0.30	−0.03 to 0.62	NS	−0.05	−0.53 to 0.42	NS	0.24	−0.15 to 0.64	NS
	Female	All	0.14	−0.13 to 0.41	NS	0.11	−0.28 to 0.50	NS	0.25	−0.07 to 0.58	NS
		≥ 65	0.17	−0.14 to 0.47	NS	0.22	−0.23 to 0.66	NS	0.38	0.02 to 0.75	*

Abbreviations: CI, confidence interval; NS, not significant.

a Change in ER per 10-μg/m^3^ increase in concentration of air pollutant averaged over the lag 0 and lag 1 days when influenza intensity changed from the baseline (0%) to the mean level (10%).

b ER per 10-μg/m^3^ increase in concentration of air pollutant averaged over the lag 0 and lag 1 days when influenza intensity is assumed equal to 0.

c ER per 10-μg/m^3^ increase in concentration of air pollutant averaged over the lag 0 and lag 1 days when intensity is assumed equal to 10% (mean level of influenza intensity).

* 0.01 < *p* ≤ 0.05;

** 0.001 < *p* ≤ 0.01;

# *p* ≤ 0.001.
